# Impact of COVID‐19 on Respiratory Virus Infections in Children, Japan, 2018–2023

**DOI:** 10.1002/iid3.70176

**Published:** 2025-03-12

**Authors:** Emi Takashita, Kohei Shimizu, Chiharu Kawakami, Tomoko Momoki, Miwako Saikusa, Hiroki Ozawa, Makoto Kumazaki, Shuzo Usuku, Nobuko Tanaka, Ryuichi Senda, Ichiro Okubo, Seiichiro Fujisaki, Shiho Nagata, Hiroko Morita, Hideka Miura, Kayo Watanabe, Mina Nakauchi, Yoko Matsuzaki, Shinji Watanabe, Hideki Hasegawa, Yoshihiro Kawaoka

**Affiliations:** ^1^ Research Center for Influenza and Respiratory Viruses, National Institute of Infectious Diseases Tokyo Japan; ^2^ Yokohama City Institute of Public Health Kanagawa Japan; ^3^ Pandemic Preparedness, Infection, and Advanced Research Center The University of Tokyo Tokyo Japan; ^4^ Research Center for Global Viral Diseases National Center for Global Health and Medicine Research Institute Tokyo Japan; ^5^ Yamagata University Faculty of Medicine Yamagata Japan; ^6^ Influenza Research Institute, Department of Pathobiological Sciences, School of Veterinary Medicine University of Wisconsin‐Madison Madison Wisconsin USA; ^7^ Division of Virology, Institute of Medical Science The University of Tokyo Tokyo Japan

**Keywords:** COVID‐19, influenza, respiratory syncytial virus, rhinovirus, SARS‐CoV‐2, viral interference

## Abstract

**Background:**

COVID‐19, caused by SARS‐CoV‐2, was first documented in Japan in January 2020. We previously reported an increased risk of rhinovirus infections among children during the early phase of the COVID‐19 pandemic. Here, we assessed the impact of COVID‐19 on respiratory virus infections after SARS‐CoV‐2 spread nationwide.

**Methods:**

We analyzed clinical specimens from 4012 patients with respiratory infections in Yokohama, Japan from January 2018 to April 2023.

**Results:**

Among 15 representative respiratory viruses we detected (influenza virus, rhinovirus, coxsackievirus, echovirus, enterovirus, human coronavirus 229E, HKU1, NL63, and OC43, human metapneumovirus, human parainfluenza virus, human parechovirus, RSV, human adenovirus, human bocavirus, human parvovirus B19, herpes simplex virus type 1, and varicella‐zoster virus), influenza was most affected by the COVID‐19 pandemic, with no influenza viruses detected for nearly 3 years.

**Conclusions:**

The decrease in influenza infections following the emergence of SARS‐CoV‐2 may have contributed to the previously reported increase in rhinovirus infections. The rhinovirus outbreak, rather than SARS‐CoV‐2, may have contributed to the decrease in enveloped virus infections (RSV, parainfluenza viruses, metapneumovirus, and coronavirus 229E, HKU1, NL63, and OC43), possibly due to negative virus‐virus interactions.

## Graphical abstract

The increased rhinovirus prevalence, rather than SARS‐CoV‐2, may have contributed to the decrease in enveloped virus infections, possibly due to negative virus‐virus interactions.

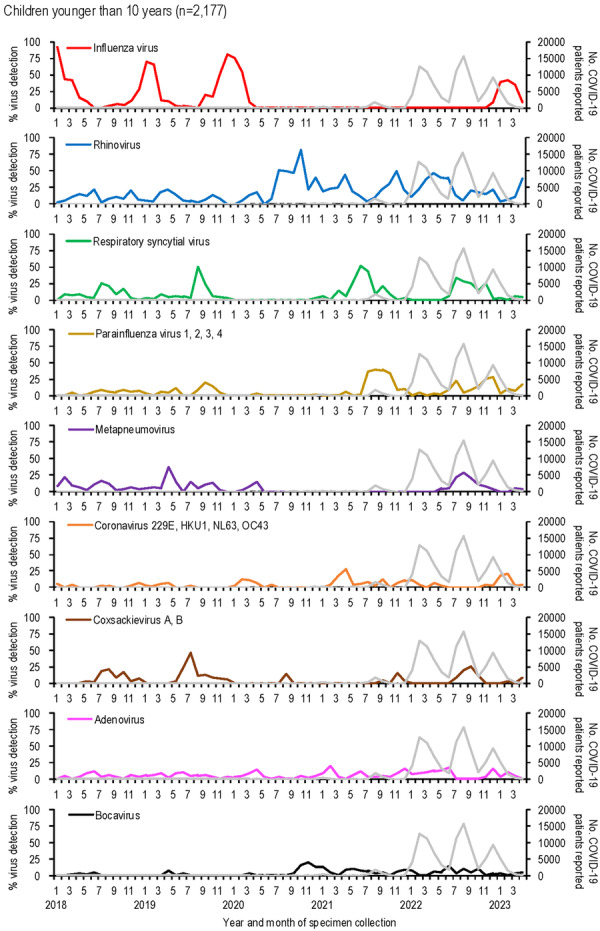

## 1 Introduction

Severe acute respiratory syndrome coronavirus 2 (SARS‐CoV‐2), which causes coronavirus disease 2019 (COVID‐19), was first detected in Japan in January 2020, and then spread throughout the country [1]. The pandemic drastically changed the epidemiology of respiratory viruses, particularly in pediatric populations. For example, in 2021, we reported an increased risk of rhinovirus infections among children younger than 10 years during the early phase of the COVID‐19 pandemic [2]. In that previous study, we analyzed clinical specimens from 2244 patients with respiratory infections in Yokohama, Japan, collected between January 2018 and September 2020. We saw a sizeable decrease in the detection rate of influenza and other respiratory viruses (coxsackievirus; echovirus; enterovirus; human coronavirus 229E, HKU1, NL63, and OC43; human metapneumovirus; human parainfluenza virus; human parechovirus; human respiratory syncytial virus (RSV); human adenovirus; human bocavirus; human parvovirus B19; herpes simplex virus type 1; and varicella‐zoster virus) among all patients after the emergence of SARS‐CoV‐2. In contrast, the detection rate of rhinovirus increased appreciably in children younger than 10 years but not in patients aged 10 years or older [2]. During this period, SARS‐CoV‐2 infections were less frequent in children than in adults, and COVID‐19 had not extensively spread among children [2].

This difference in detection rates is likely the results of viral interference, as well as differences in immune response and in the effects of public health interventions. Viral interference affects respiratory viral infections at the host and population levels [3, 4]. Respiratory viruses can circulate concurrently or sequentially, leading to virus–virus interactions. Homologous viral interaction, such as that between influenza viruses of different subtypes or lineages, induces cross‐reactive immunity and lowers the risk of secondary infections [5]. Heterologous viral interaction (e.g., between SARS‐CoV‐2 and influenza viruses) relies on nonspecific innate immune responses induced by the first virus suppressing the replication of subsequent viruses [3].

Viral interference between SARS‐CoV‐2 and other respiratory viruses, including influenza A(H1N1)pdm09, A(H3N2), or B/Victoria‐lineage viruses has been reported [6, 7, 8, 9, 10, 11, 12, 13]. Moreover, there have been reports of changes in RSV subtype predominance since the emergence of SARS‐CoV‐2 [14]. Yet the long‐term effects of COVID‐19 on the dynamics of respiratory viruses in pediatric populations have not been well studied.

Accordingly, here, we examined clinical specimens from 4012 patients with respiratory infections in Yokohama, Japan, collected between January 2018 and April 2023. We analyzed the detection rates and genotypes of influenza virus, rhinovirus, and RSV to assess the effect of COVID‐19 on respiratory virus infection rates in children after SARS‐CoV‐2 spread throughout Japan.

## 2 Methods

### 2.1 Clinical Specimens

Respiratory specimens from patients with influenza‐like illnesses were collected throughout the year at sentinel sites, which included internal medicine and pediatric locations, as part of the National Epidemiological Surveillance of Infectious Diseases. After the emergence of SARS‐CoV‐2, specimens from suspected COVID‐19 cases were collected at designated medical institutions for COVID‐19 under the Active Epidemiological Investigation for COVID‐19. Nasal swabs, throat swabs, nasal discharge, saliva, tracheal aspiration fluid, or sputum were obtained from 4012 patients in Yokohama, Japan between January 2018 and April 2023. Diagnoses included upper or lower respiratory tract infection, viral infection, viral exanthema, or infectious gastroenteritis. We excluded patients with confirmed SARS‐CoV‐2 infections from the study. Of the 4012 patients, 2078 (51.8%) were male, 1931 (48.1%) were female, and 3 (0.1%) provided no sex information; 2177 (54.3%) were younger than 10 years, 1313 (32.7%) were 10–59 years, 500 (12.5%) were aged 60 years or older, and 22 (0.5%) did not provide age information. We did not seek ethical approval because we analyzed data that was collected during routine surveillance and the Active Epidemiological Investigation for COVID‐19, authorized under Japan's Act on the Prevention of Infectious Diseases and Medical Care for Patients with Infectious Diseases. All data used in this study were anonymized.

### 2.2 Virus Detection

Influenza virus, rhinovirus, coxsackievirus types A and B, echovirus, enterovirus, human coronaviruses (229E, HKU1, NL63, and OC43), human metapneumovirus, human parainfluenza virus types 1–4, human parechovirus, RSV, human adenovirus, human bocavirus, human parvovirus B19, herpes simplex virus type 1, and varicella‐zoster virus were identified by using virus isolation, PCR, RT‐PCR, real‐time RT‐PCR, Sanger sequencing, FTD Respiratory pathogens 21 (Fast Track Diagnostics, Sliema, Malta), and Seeplex RV15 OneStep ACE Detection (Seegene, Seoul, Republic of Korea) as previously described [2]. The DNA viruses in this analysis included human adenovirus, human bocavirus, human parvovirus B19, herpes simplex virus type 1, and varicella‐zoster virus. All other viruses in this analysis were RNA viruses.

### 2.3 Genotyping of Influenza Virus, Rhinovirus, and RSV

Influenza A, B, and C virus genotypes were determined by using real‐time RT‐PCR as previously described [15, 16, 17]. Rhinovirus A, B, and C were identified by using next‐generation sequencing and BLAST and phylogenetic analyses as previously described [18]. Reference sequences from GenBank for BLAST and phylogenetic analyses: DQ473503, DQ473487, DQ473488, DQ473493, EF582385, EF582386, GQ223228, GU219984, JF317015, KF688606, KY369883, MF775366, MK520815, MN369038, MW969530, MZ153252, MZ153277, MZ268677, MZ363416, MZ363449, MZ629123, MZ629160, OK161404, OK254858, OK649395, OK649409, OM001382, and OM001412. RSV A and B genotypes were determined by using real‐time RT‐PCR or Sanger sequencing as previously described [19].

## 3 Results

### 3.1 Detection of Respiratory Viruses by Age Group From January 2018 to April 2023

The numbers of respiratory viruses detected and COVID‐19 cases in Yokohama, Japan, from January 2018 to April 2023, are shown in Figure 1 and the Supplementary table. The first COVID‐19 case in Yokohama was reported in February 2020, with subsequent peaks occurring every 4–7 months (Figure 1A). Among the 4012 specimens analyzed, influenza virus was the most frequently detected virus (*n* = 668), followed by rhinovirus (*n* = 484) and RSV (*n* = 229) (Figure 1A). Co‐detection of two or more viruses was observed in 191 cases. Figure 1B–D illustrate the numbers of viruses detected by age group, showing that children younger than 10 years experienced the highest detections for most viruses, except for influenza in the 10‐ to 59‐year‐old group. Due to the limited numbers of viruses detected in older age groups, further analysis focused on children younger than 10 years.

Figure 1The number of COVID‐19 patients reported and the number of respiratory viruses detected by age group from January 2018 to April 2023 in Yokohama, Japan. All patients (A; *n* = 4012), children younger than 10 years (B; *n* = 2177), patients aged 10–59 years (C; *n* = 1313), and patients aged 60 years or older (D; *n* = 500). Representative respiratory viruses with maximum monthly values of 5 or more are shown. Parentheses next to the virus name indicate the total number of viruses detected. The gray line indicates the number of COVID‐19 patients reported by the local government. Age information was not available for 22 of 4012 patients.

**Figure:**
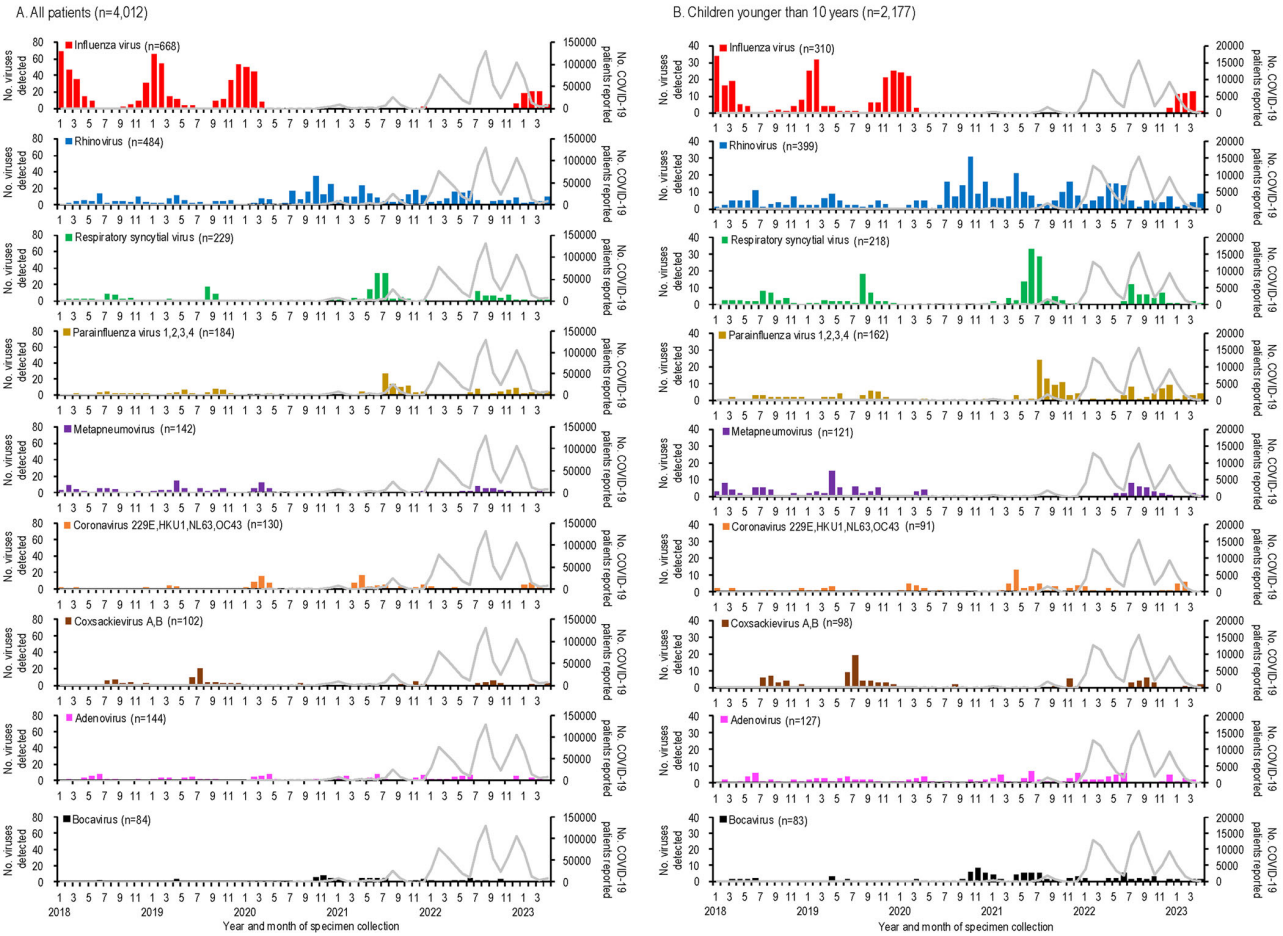

**Figure:**
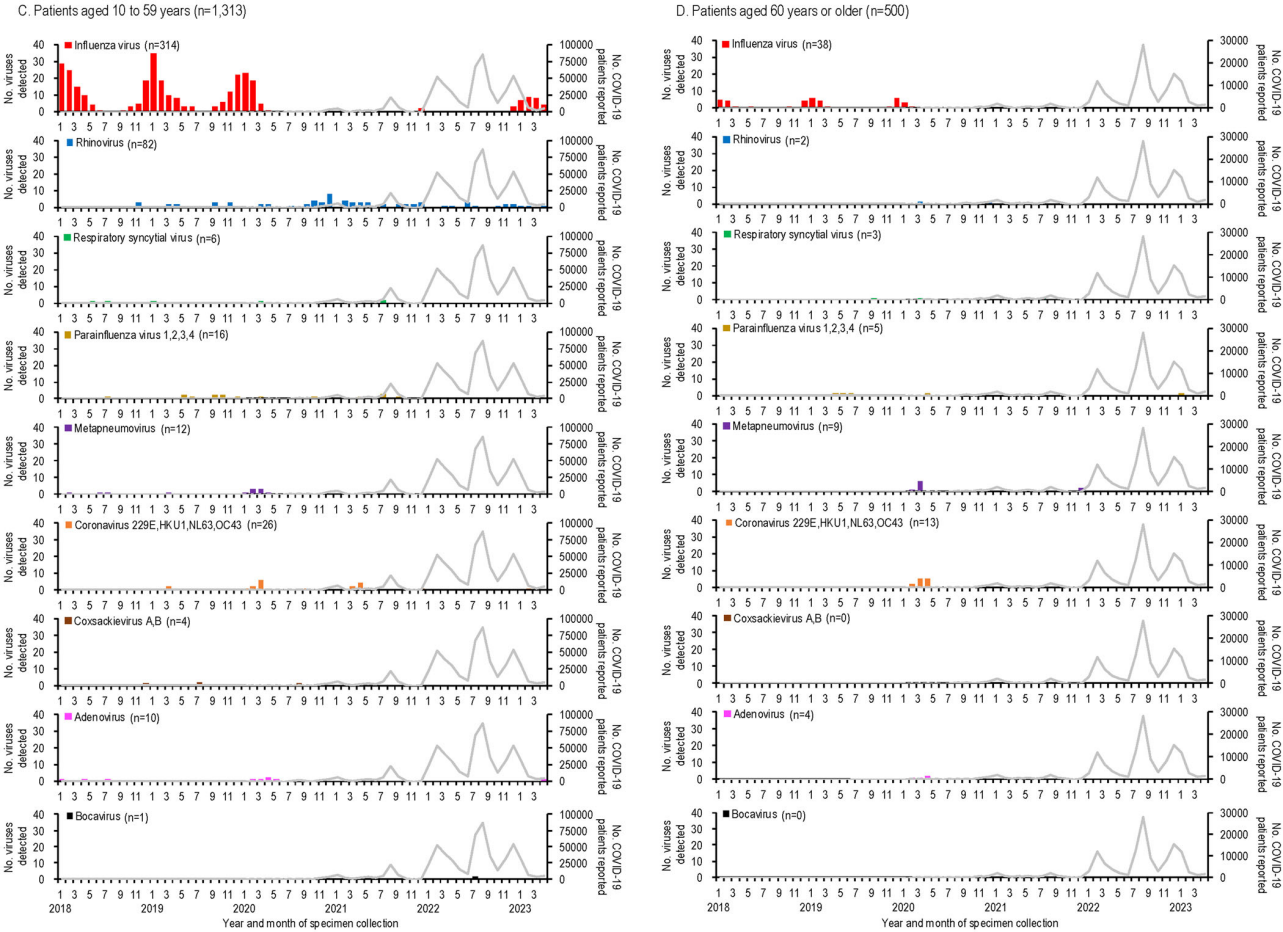

### 3.2 Respiratory Virus Activities in Children Younger Than 10 Years From January 2018 to April 2023

**Figure 2:**
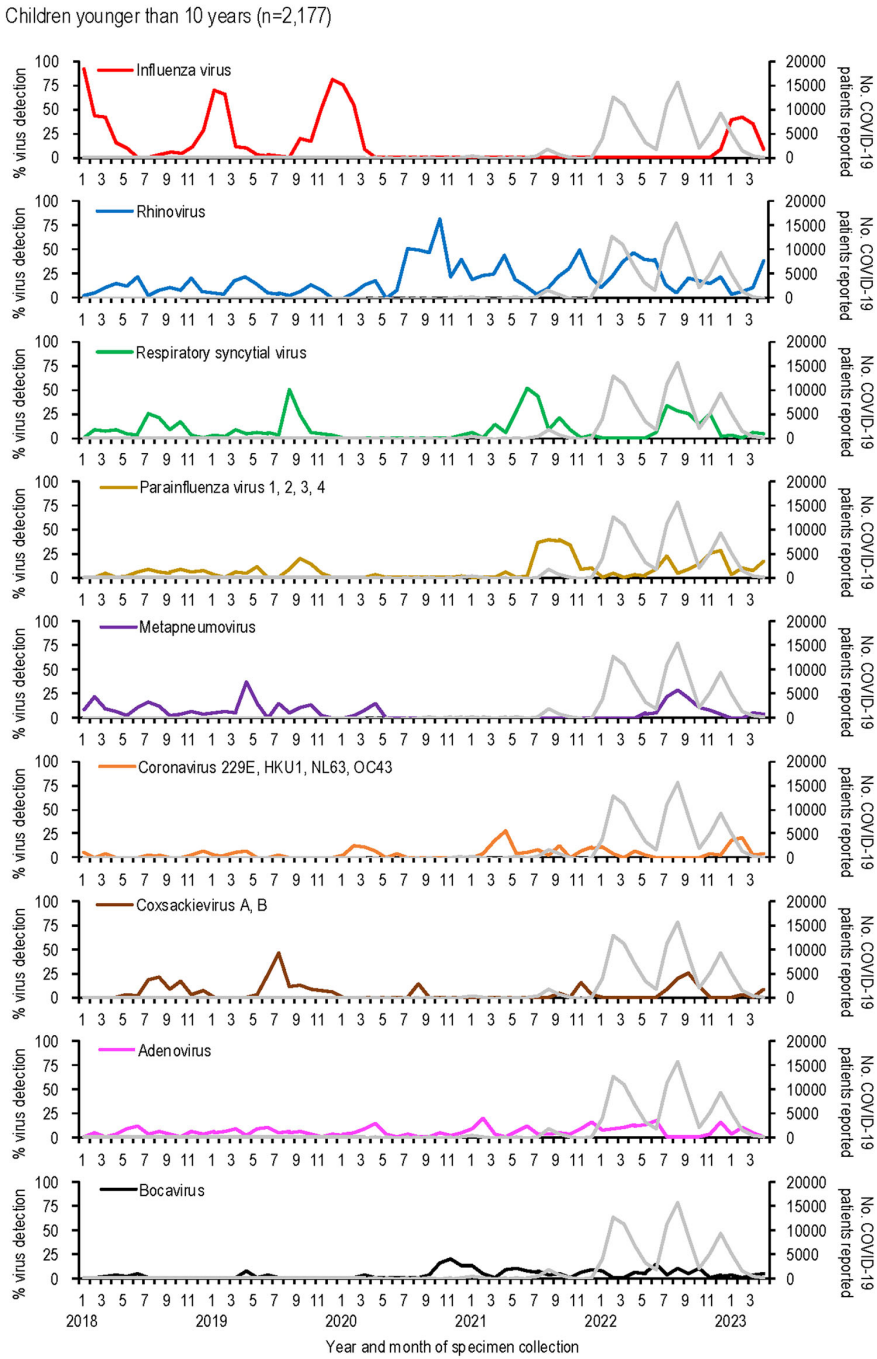
The number of COVID‐19 patients reported and the detection rate of respiratory viruses in children younger than 10 years from January 2018 to April 2023 in Yokohama, Japan. The detection rate for each virus was calculated as the number of detections per analyses per month. Representative respiratory viruses with a maximum monthly detection rate of 10% or more are shown. The gray line indicates the number of COVID‐19 patients reported by the local government.

The detection rates of respiratory viruses in children younger than 10 years are shown in Figure 2. Influenza virus, which typically peaked each winter before the emergence of SARS‐CoV‐2, was undetectable for 32 months, from April 2020 to November 2022. A resurgence occurred in February 2023, with a detection peak of 42.9%, lower than pre‐pandemic peaks (e.g., 91.9% in January 2018, 69.4% in January 2019, and 80.6% in December 2019).

Rhinovirus activity increased during the pandemic, with peaks reaching 44.7%–81.6% during periods when influenza was absent (autumn 2020 to spring 2022), compared to pre‐pandemic peaks of 13.9%–22.5%. RSV activity, which typically peaked in the summer [20], was absent from January to November 2020 but resumed normal seasonal patterns by 2021.

Other viruses, such as parainfluenza virus and metapneumovirus, showed reduced activity early in the pandemic. Detection rates for these viruses increased two‐fold post‐pandemic: parainfluenza virus rates rose from 20.0% in September 2019 to 39.4% in August 2021, while metapneumovirus rates rose from 14.3% in April 2020 to 28.6% in August 2022.

Human coronaviruses (229E, HKU1, NL63, and OC43) showed lower detection numbers and rates compared to other enveloped viruses, such as influenza virus, RSV, parainfluenza viruses, and metapneumovirus (Figures 1, 2). Their activity was less noticeably impacted by the emergence of SARS‐CoV‐2. During the early phase of the COVID‐19 pandemic, no human coronaviruses were detected for 7 months (July 2020–January 2021), and their detection rates declined further as COVID‐19 cases increased.

Among non‐enveloped viruses, coxsackievirus A and B activity peaked in summer (July–August) before the pandemic but shifted to autumn (September–November) after SARS‐CoV‐2 emerged. Adenovirus was consistently detected throughout the study period, showing no significant impact from SARS‐CoV‐2. In contrast, human bocavirus detections and detection rates increased notably following the spread of SARS‐CoV‐2 (Figures 1, 2).

### 3.3 Genotypes of Influenza Virus, Rhinovirus, and RSV Detected in Children Younger Than 10 Years From January 2018 to April 2023

**Figure 3:**
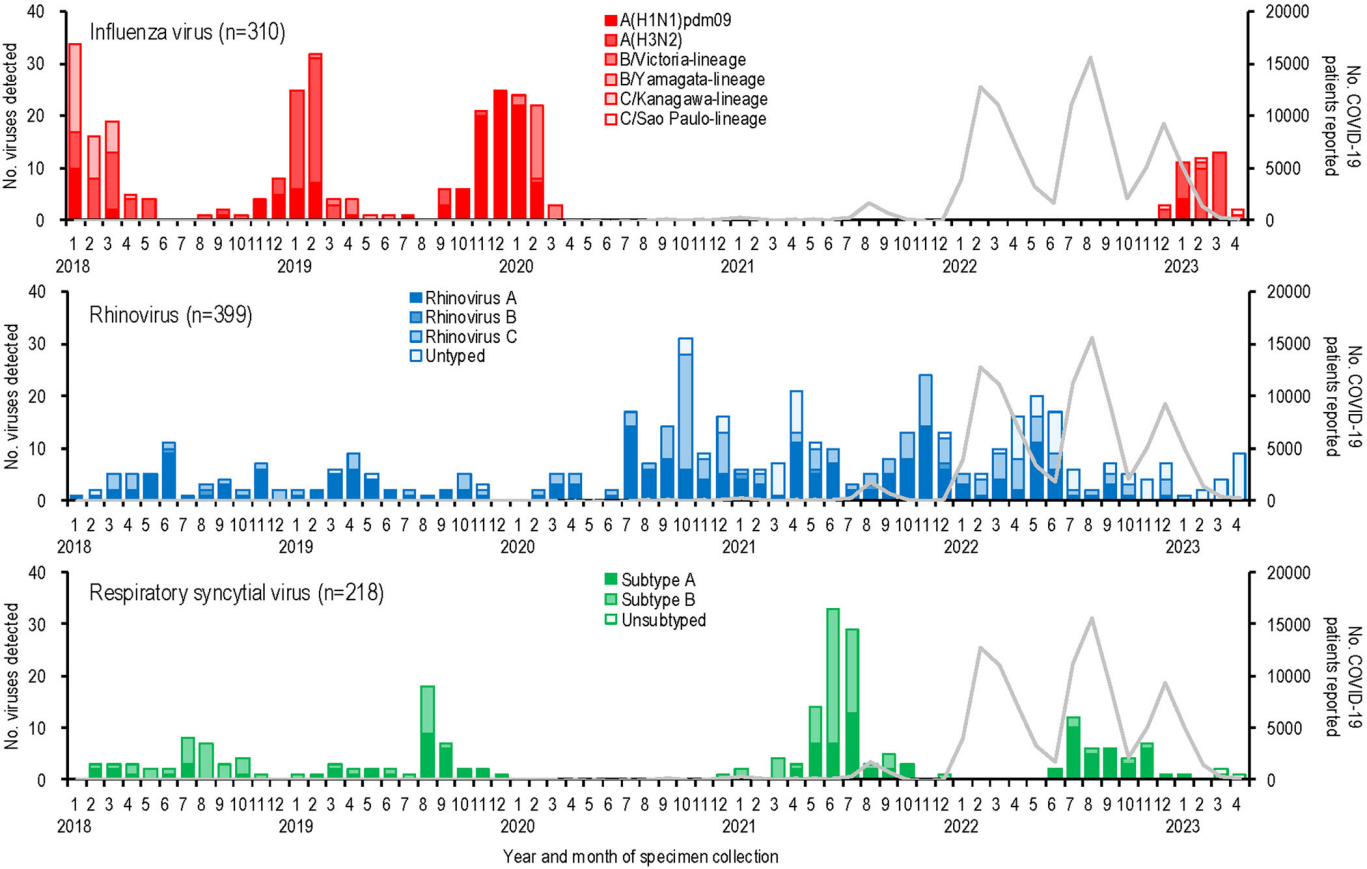
The number of COVID‐19 patients reported and the genotypes of influenza virus, rhinovirus, and respiratory syncytial virus detected in children younger than 10 years from January 2018 to April 2023 in Yokohama, Japan. The number of viruses of each genotype is shown. Parentheses next to the virus name indicate the total number of viruses detected. The gray line indicates the number of COVID‐19 patients reported by the local government.

Lastly, we analyzed the genotypes of the most frequently circulating viruses—influenza virus, rhinovirus, and RSV—detected in children younger than 10 years (Figure 3). During the 2017–18 season, influenza A(H3N2) and B/Yamagata‐lineage viruses co‐circulated. A(H3N2) became predominant in the 2018–19 season, followed by A(H1N1)pdm09 in the 2019–20 season. Influenza activity was absent during the 2020–21 and 2021–22 seasons, coinciding with the spread of SARS‐CoV‐2. In the 2022–23 season, A(H3N2) predominated, with additional detections of A(H1N1)pdm09, B/Victoria‐lineage viruses, and rare influenza C viruses. Notably, three of the four influenza C viruses were detected in the 2022–23 season.

Rhinovirus genotyping revealed consistent co‐circulation of rhinovirus A and C throughout the study period, with only sporadic detections of rhinovirus B in four patients. This suggests that the emergence of SARS‐CoV‐2 had little to no impact on the genetic diversity of rhinovirus.

RSV subtype analysis showed alternating predominance, with subtype B prevailing in 2018 and 2021 and subtype A dominating in 2019 and 2022. No RSV outbreaks occurred in 2020 during the early COVID‐19 pandemic. From 2018 to 2021, subtype B detections were 2–3 times higher than subtype A. However, after SARS‐CoV‐2 spread, subtype A detections increased markedly, outnumbering subtype B by a 7:1 ratio in 2022. This shift aligns with findings from Australia [14].

## 4 Discussion

To evaluate the impact of COVID‐19 on respiratory virus infections following the spread of SARS‐CoV‐2, we analyzed clinical specimens from 4012 patients with respiratory infections in Yokohama, Japan, collected between January 2018 and April 2023. Among the 15 representative respiratory viruses detected, the emergence of SARS‐CoV‐2 had the most significant impact on influenza infections.

The first COVID‐19 case in Yokohama was reported in February 2020, after which the detection rate of influenza virus declined dramatically. No influenza viruses were detected for nearly 3 years (Figure 1B). This decline can be attributed to public health measures such as mask‐wearing, physical distancing, hand hygiene [21], and Japan's strict border controls implemented from February 2020 to October 2022. These measures likely blocked the introduction of influenza viruses from overseas. Influenza re‐emerged during the 2022–2023 season, predominantly with A(H3N2) virus, coinciding with the lifting of border restrictions.

Non‐enveloped viruses, such as coxsackievirus, adenovirus, and bocavirus, were less affected during the pandemic, likely due to their resistance to ethanol‐based disinfectants and environmental stability [22, 23]. In contrast, rhinovirus infections increased significantly in children younger than 10 years, likely due to its intrinsic stability and possible viral interference.

Enveloped viruses, including RSV, parainfluenza viruses, metapneumovirus, and human coronaviruses, showed markedly decreased activity during the early phase of the pandemic, with no detections for 7–24 months (Figures 1, 2). While the circulation of these viruses decreased, rhinovirus became more dominant, suggesting viral interference. At the population level, behavioral changes such as school closures may have influenced virus circulation. At the host level, rhinovirus infection likely induced interferon‐stimulated genes (ISGs), inhibiting the replication of other viruses [24, 25, 26]. In contrast, SARS‐CoV‐2 infection triggers limited ISG responses but elevated chemokine expression [27], potentially giving rhinovirus a competitive advantage.

Immune responses between adults and children also differ significantly. Children rely more on innate immunity, including higher natural killer cell activity and cytokine responses, which protect them from severe infections [28]. Reduced SARS‐CoV‐2 binding affinity to the ACE2 receptor in children, coupled with potential cross‐immunity from seasonal coronaviruses, may have contributed to lower SARS‐CoV‐2 infection rates and distinct respiratory virus patterns during the pandemic [28].

Genotyping of influenza virus, rhinovirus, and RSV highlighted the impact of COVID‐19 on viral evolution (Figure 3). Influenza activity was absent during the 2020–21 and 2021–22 seasons but re‐emerged with A(H3N2) and influenza C viruses predominating in the 2022–23 season. The B/Yamagata‐lineage virus has not been detected since 2019. Rhinovirus genetic diversity remained stable, but RSV subtype predominance shifted, with subtype A becoming more dominant after SARS‐CoV‐2 spread. This shift, also observed in Australia [14], may reflect changes in viral interference or population immunity.

This study has two key limitations. First, it was conducted in a single region, potentially limiting the generalizability of our findings. Second, the retrospective collection of data may have introduced biases related to sample availability and testing methodologies. Multi‐region, longitudinal studies are needed to further understand the long‐term impact of the COVID‐19 pandemic on respiratory virus epidemiology, particularly in children. Such studies should also investigate variations in virus dynamics across regions and healthcare settings, as well as the mechanisms underlying viral interference between SARS‐CoV‐2 and other respiratory viruses.

The Active Epidemiological Investigation for COVID‐19 in Japan ended in May 2023, transitioning COVID‐19 surveillance to the National Epidemiological Surveillance of Infectious Diseases, akin to influenza. Consequently, the study period was set to April 2023. Since then, only the number of COVID‐19 cases per sentinel has been reported. However, SARS‐CoV‐2 continues to evolve, accumulating amino acid substitutions in its proteins, and the outbreak persists in Japan. To monitor ongoing trends, respiratory virus surveillance has continued since May 2023 using sentinel‐based reporting.

## 5 Conclusions

Our study investigated how the COVID‐19 pandemic affected the rates of other respiratory virus infections in children. We found that, during the pandemic, the incidence of influenza and other enveloped virus infections was significantly reduced, likely as a result of viral interference and public health measures. The reduced influenza activity also led to an increase in the rates of infections caused by non‐enveloped viruses such as rhinovirus.

By improving our understanding of the complex interplay between viral infections and analyzing the consequences of public health interventions, we can develop better strategies to combat co‐circulating respiratory viruses, particularly in vulnerable populations such as children.

## Author Contributions

**Emi Takashita:** conceptualization, investigation, methodology, writing – original draft. **Kohei Shimizu:** investigation, writing – review and editing. **Chiharu Kawakami:** investigation, writing – review and editing. **Tomoko Momoki:** investigation, writing – review and editing. **Miwako Saikusa:** investigation, writing – review and editing. **Hiroki Ozawa:** investigation, writing – review and editing. **Makoto Kumazaki:** investigation, writing – review and editing. **Shuzo Usuku:** investigation, writing – review and editing. **Nobuko Tanaka:** writing – review and editing. **Ryuichi Senda:** writing – review and editing. **Ichiro Okubo:** writing – review and editing. **Seiichiro Fujisaki:** investigation, writing – review and editing. **Shiho Nagata:** investigation, writing – review and editing. **Hiroko Morita:** investigation, writing – review and editing. **Hideka Miura:** investigation, writing – review and editing. **Kayo Watanabe:** investigation, writing – review and editing. **Mina Nakauchi:** investigation, writing – review and editing. **Yoko Matsuzaki:** investigation, writing – review and editing. **Shinji Watanabe:** writing – review and editing. **Hideki Hasegawa:** writing – review and editing. **Yoshihiro Kawaoka:** conceptualization, writing – review and editing.

## Conflicts of Interest

The authors declare no conflicts of interest.

## Supporting information

Supporting information.

## Data Availability

The data that supports the findings of this study are available in the supporting material of this article.
